# Resistance to Thyroid Hormone in Crohn's Disease: A Clinical Challenge of Refractory Thyroid Stimulating Hormone Elevation and Therapeutic Dilemmas

**DOI:** 10.1002/ccr3.71015

**Published:** 2025-09-26

**Authors:** Ahmad Al‐Bitar, Dana Al‐Masalma, Moumen Alkarkoukly, Mohammad Al‐Hawasli

**Affiliations:** ^1^ Faculty of Medicine Damascus University Damascus Syrian Arab Republic; ^2^ Endocrine and Metabolism Department Damascus Hospital Damascus Syrian Arab Republic

**Keywords:** case report, Crohn's disease, inflammatory bowel disease, Refetoff syndrome, thyroid hormone resistance

## Abstract

Thyroid hormone resistance (RTH) is a rare genetic disorder characterized by diminished responsiveness of target tissues to thyroid hormones, primarily due to mutations in the thyroid hormone receptor‐beta (TR‐β) gene. This condition could lead to an increased level of Thyroid stimulating hormone (TSH) and varying degrees of hypothyroid symptoms, despite normal or elevated levels of circulating thyroid hormones. Concurrently, Crohn's disease (CD), an inflammatory bowel disease, poses significant diagnostic and therapeutic challenges due to its multifactorial etiology and association with various autoimmune disorders. This case report describes a 30‐year‐old female patient diagnosed with both RTH and CD. Given a history of thyroidectomy, the patient was on Levothyroxine supplement and also was taking corticosteroids for Crohn's disease. Despite adherence to therapy, persistently elevated TSH levels were noted, and the patient still exhibited signs of hypothyroidism. Altogether, it raised suspicions for RTH. After 1 year, we successfully managed her Crohn's disease, achieving sustained remission. Surprisingly, clinical and biochemical markers of hypothyroidism also showed significant improvement. This case illustrates the intricate relationship between autoimmune conditions and thyroid dysfunction, emphasizing the necessity for comprehensive evaluation in patients with overlapping symptoms. The findings highlight the importance of recognizing RTH in patients with unexplained thyroid abnormalities, particularly in the context of coexisting inflammatory bowel diseases, to optimize management and improve patient outcomes.

**Summary**

- In patients with inflammatory bowel disease (IBD) and unexplained thyroid dysfunction—particularly elevated TSH despite adequate levothyroxine dosing—consider resistance to thyroid hormone (RTH) alongside malabsorption.
- Corticosteroids, while treating Crohn's inflammation, may unmask RTH by resolving malabsorption and normalizing thyroid labs.
- Systematic thyroid evaluation (TSH, FT4, genetic testing) is critical in refractory IBD to avoid therapeutic delays.

## 1 Introduction

Thyroid hormones regulate the metabolic activity of most body organs, making thyroid diseases common. In iodine‐sufficient communities, spontaneous hypothyroidism occurs in 1%–2% of the population, with women being 10 times more affected, especially older women. The thyroid gland produces hormones that impact nearly every organ and tissue. The body primarily releases the inactive form, thyroxine (T4), while the active form, triiodothyronine (T3), is released in smaller amounts. Thyroid hormones critically control functions in all organs, including the GI tract, affecting motility and digestive juice secretion. Both hypo‐ and hyperthyroidism can disrupt GI motility and function [1].

RTH is an uncommon syndrome inherited dominantly where the autosomes are involved, resulting from a single mutation in the thyroid hormone receptor‐beta (TR‐β) gene. It is estimated that around 1 in 40,000 live births are affected by resistance to thyroid hormone. This condition was first documented by Refetoff et al. [2].

The symptoms observed in RTH can vary significantly, encompassing a wide spectrum from issues like hypothyroidism, short stature, and impaired intellectual development to hyperthyroidism, goiter, and tachycardia [3].

Crohn's disease (CD) is a chronic inflammatory bowel disease (IBD) marked by transmural inflammation affecting various parts of the gastrointestinal tract. Its incidence and prevalence vary by region, with the highest rates in Europe, Oceania, and North America. Newly industrialized countries in Asia, Africa, and South America are also experiencing rising incidence rates, highlighting a global challenge. CD peaks between ages 20 and 30, with common symptoms including diarrhea, abdominal pain, fatigue, and weight loss, as well as extra‐intestinal issues like arthropathy and skin or eye problems [4].

Ulcerative colitis (UC) and Crohn's disease (CD) stand as the prevailing inflammatory bowel diseases (IBD). Both UC and CD manifest as persistent conditions marked by recurrent episodes, featuring gut inflammation attributed to factors like the environment or immunological triggers [5].

Thyroid disorders are noted to be more prevalent among individuals with ulcerative colitis (UC) and Crohn's disease (CD) in contrast to the general population [6].

Historically, many questions arose regarding the connection between thyroid disorders and gastrointestinal diseases. We now present a case of an adult woman with Resistance to Thyroid Hormone (RTH) who is also affected by Crohn's disease, prompting us to reflect on this coexistence.

## 2 Case History

A 30‐year‐old female patient presented to the endocrine department with hyperthyroidism symptoms, including tachycardia, agitation, tremor, palpitation, and dyspnea, despite undergoing a total thyroidectomy after thyroid gland enlargement and hypothyroidism symptoms with multiple nodules and was subsequently placed on oral Levothyroxine at a daily dosage of 300 μg. However, the thyroid panel revealed an elevated TSH level of 55 μIU/L and a normal FT4 level of 0.94 ng/dL.

Vital signs and physical examination were unremarkable without any abnormal findings. There was no family history of autoimmune diseases, thyroid diseases, or other significant illnesses.

The patient had a history of hiatal hernia and gastroesophageal reflux disease (GERD) and underwent laparoscopic Nissen fundoplication. The patient developed severe gastrointestinal symptoms (including abdominal pain and diarrhea) accompanied by extraintestinal manifestations such as joint pain, eye redness, and elevated liver enzymes. The lower endoscopy revealed a 3‐cm ulcer at the terminal ileum's beginning. The microscopic examination of biopsies taken from the colon and terminal ileum showed:

- Ulceration and severe inflammatory infiltration in the lamina propria of the terminal ileum mucosa. The infiltrate was composed of plasma cells and lymphocytes. Granulomas composed of giant and epithelioid cells were present without necrosis.
- Colonic mucosa with mild pleomorphic inflammatory infiltrate comprising neutrophils, macrophages, plasma cells, and lymphocytes, and no malignancy was noted.

## 3 Methods

These findings, in correlation with clinical presentation, led to a diagnosis of Crohn's disease, and the patient was prescribed prednisolone 200 mg.

An elevated TSH level and a normal FT4 level after total thyroidectomy coexisting with Crohn's disease suggest either malabsorption of oral levothyroxine due to Crohn's disease or thyroid hormone resistance syndrome.

The corticosteroids induced the remission of Crohn's disease, improved the thyroid panel, and eliminated the malabsorption from differential diagnosis, and this highly suggests the diagnosis of resistance to thyroid hormone (RTH) syndrome.

## 4 Conclusion and Results

After 1 year of coherent intake of levothyroxine 250 μg and prednisolone 200 mg, her lab results show a remarkable improvement in both the symptoms and the lab results (TSH level of 28 μIU/L, FT4 level of 1.1 ng/dL).

In this case, we presented a 30‐year‐old female patient with a complex interplay of hypothyroidism and Crohn's disease, highlighting the challenges in diagnosing and managing concurrent autoimmune conditions. Despite undergoing total thyroidectomy and receiving appropriate levothyroxine therapy, the patient's persistent elevated TSH levels suggested the possibility of thyroid hormone resistance syndrome (THR).

The subsequent diagnosis of Crohn's disease, characterized by significant gastrointestinal symptoms and histological findings, revealed a potential mechanism for malabsorption affecting the patient's thyroid medication. The initiation of corticosteroid therapy not only induced remission of Crohn's disease but also led to an improvement in thyroid function tests, further supporting the diagnosis of THR.

This case emphasizes the need for increased awareness and documentation of the association between inflammatory bowel diseases and thyroid disorders. Clinicians should remain vigilant in evaluating thyroid function in patients with IBD, as the nuances of these overlapping conditions can significantly impact treatment strategies and patient outcomes. Further research is warranted to elucidate the underlying mechanisms linking these disorders and to enhance our understanding of their clinical implications.

## 5 Discussion

Many studies indicate that thyroid disorders are more common in patients with inflammatory bowel diseases (IBD) than in other populations [5, 6, 7].

However, studies that correspond to this scientific theory and record the accompanying cases are not enough [5, 6, 7].

Furthermore, the relationship between IBD and thyroid disorders is not sufficiently documented.

In our case, we have a 30‐year‐old woman who suffered in 2019 from hypothyroidism symptoms and multiple thyroid nodules. A year later, she underwent a total thyroidectomy and was put on medication treatment. Despite the drug treatment, thyroid function tests showed normal FT4 levels and elevated TSH levels. With additional investigation means and tests and after the dismissal of other differential diagnoses, she was diagnosed with thyroid hormone resistance syndrome (THR).

THR is a syndrome where the body's tissue becomes resistant to the thyroid hormone effect [8]. It is considered a rare condition that is often misdiagnosed or difficult to diagnose [8].

It has three patterns that differ according to the thyroid hormone‐resistant tissue, so resistance is at the pituitary gland with or without resistance to peripheral tissues [9]. In the case of our patient, it is a second type where it suffers from resistance of the pituitary gland to the thyroid hormone, which means it responds to the hormone through the peripheral receptors, while the pituitary receptors remain unresponsive. This results in continuous high TSH levels and being unresponsive to treatment. In 2023, the patient suffered from digestive symptoms, including abdominal pain, diarrhea, joint pain, and redness of the eye. A lower endoscopy was performed and she was diagnosed with Crohn's disease, a multi‐factor inflammatory bowel disease that may be the result of many environmental and immunological factors [10]. Furthermore, Crohn's disease is a chronic inflammatory disease affecting various areas of the intestinal digestive tract [4]. Crohn's is considered a widespread disease in the world, but Europe is ranked first by infection [11]. Diagnosis involves the integration of clinical symptoms with laboratory analysis and endoscopy [12]. Recent studies have shown that many immune and genetic factors form the cornerstone of Crohn's disease [13, 14]. Since THR syndrome has genetic mutations and is sometimes associated with immune thyroid diseases, the immune causes behind the two diseases can play a significant role in their combination [13, 14, 15].

According to previous studies, the results of our study correspond to the results of these studies because IBD patients have a higher incidence of thyroid disorders than others.

This makes it necessary to recommend the laboratory's analysis of thyroid functions in patients with IBD when observing resistance to treatment, thyroid enlargement, or any thyroid function disorder. Also, we need to document these cases. We may find a deeper understanding of the unknown factors that cause the combination of these diseases. It will be important to gather additional information about these cases.

## Author Contributions

**Ahmad Al‐Bitar:** formal analysis, investigation, methodology, writing – original draft, writing – review and editing. **Dana Al‐Masalma:** investigation, writing – original draft. **Moumen Alkarkoukly:** writing – original draft. **Mohammad Al‐Hawasli:** supervision, writing – review and editing.

## Ethics Statement

The authors have nothing to report.

## Consent

A written informed consent was obtained from the patient.

## Conflicts of Interest

The authors declare no conflicts of interest.

## Data Availability

Data available on request from the authors.
